# Banded Sleeve Gastrectomy: Better Long-Term Results? A Long-Term Cohort Study Until 5 Years Follow-Up in Obese and Superobese Patients

**DOI:** 10.1007/s11695-018-3248-2

**Published:** 2018-04-18

**Authors:** Luc Lemmens, Jelmer Van Den Bossche, Hinali Zaveri, Amit Surve

**Affiliations:** 1 Abdominal Surgery, Campus Sint-Niklaas, AZ Nikolaas, Moerlandstraat 1, 9100 Sint-Niklaas, Belgium; 2 Bariatric Medicine Institute, 1046 East 100 South, Salt Lake City, UT 84102 USA

**Keywords:** Banded sleeve gastrectomy, BLSG, Laparoscopic sleeve gastrectomy, LSG, Weight loss, Band, Silastic ring, %EWL, %EBMIL

## Abstract

**Introduction:**

The failure rate of the laparoscopic sleeve gastrectomy (LSG) is increasing. Gastric pouch dilation is frequently suggested to be one of the causes for the failure. The banded laparoscopic sleeve gastrectomy (BLSG) has been proposed to overcome this complication. This is the first study that reports the long-term outcome (> 5 years) of BLSG in obese and superobese patient population.

**Materials and Methods:**

One hundred and forty-seven patients (*n* = 51, non-banded LSG (NLSG)/*n* = 96, BLSG) were followed up for 5 years. Patients were evaluated for % excess weight loss (%EWL), % excess body mass index loss (%EBMIL), weight regain, BMI, and complications. Weight loss analysis was also done between banded and non-banded superobese patient populations.

**Result:**

There was statistical significant difference between two groups at each given time point in terms of %EWL and %EBMIL. NLSG group had higher weight loss failure rate (35.2%) and weight regain (19.6%) at the 5-year follow-up compared to BLSG group (*P* < 0.001). There was no statistical significant difference in weight loss between obese and superobese BLSG group. The complication rates were more with BLSG group (14.5%) compared to NLSG group (9.8%); no signs of band slippage, erosion, or migration were seen. There was no mortality seen.

**Conclusion:**

BLSG surgery was found to be safe and effective in maintaining weight loss on the long term compared to the NLSG group with low incidence of band-related problems. Additionally, the NLSG group had a higher rate of weight loss failure and weight regain at 5 years compared to the BLSG group.

## Introduction

Laparoscopic sleeve gastrectomy (LSG) was first reported as a part of the two-stage approach for high-risk patients undergoing laparoscopic biliopancreatic diversion duodenal switch (BPD-DS) [1]. Soon, it was recognized safe and effective as a stand-alone procedure. With its increasing popularity, it has become the most performed bariatric procedure in the world [2] and the most commonly performed bariatric procedure at US academic medical centers [3]. Technical simplicity, short operating time, high safety profile; its ability to convert, revise, or used as a staged procedure, low perioperative morbidity, and immediate calorie intake restriction are the reasons for its increasing popularity [4, 5].

With longer follow-up of the LSG, the failure rate of this procedure is also increasing [6, 7]. There is limited data on the mid-term and long-term weight loss (> 5 years and 10 years) after LSG, and thus the long-term weight loss maintenance is a major concern. Himpens et al. reported an excess weight loss (EWL) of 53% after 6 years [6]. While Alverenga et al. reported EWL of 52% at 8 years [8]. With such sobering long-term data, it is necessary for bariatric surgeons around the world to come up with the additional strategies to manage these patients.

While the cause of insufficient weight loss or weight regain is multifactorial, an increase in the gastric reservoir size due to long-term gastric pouch dilation is frequently suggested to be one of the causes [9, 10]. In case of weight loss failure, where the inadequate restriction or gastric dilation is a cause of failure, many authors proposed a safe and efficient option to increase restriction by placing an adjustable gastric band below the GE junction [11, 12].

The use of bands or rings over the gastric tube has been known previously in LRYGB, and the results have been promising [13, 14]. Banding the LSG derived from the same concept [15]. However, there is a paucity of data on banded LSG (BLSG). In this cohort study, we evaluate the long-term outcomes of BLSG and compare it with non-banded LSG (NLSG) in terms of weight loss and incidence of complications. We also describe the outcomes of BLSG between the obese and the superobese patient population at 5 years. This is the first long-term (> 5 years) report of BSLG in the literature.

## Method

Patients that had either NLSG or BLSG between May 2010 and July 2017 were analyzed for potential inclusion. All the procedures were performed by one surgeon at a single institute at the AZ Nikolaas, Belgium. Extensive information concerning pros and cons of both procedures were given to patients. Patients signed the specific consent to have the NLSG or BLSG after they made their choice. Each patient also signed the consent to have their data analyzed in a blinded fashion.

Patients were followed in a multidisciplinary program with all follow-up data entered in a programmatic database. Follow-up visit took place at 3, 6, 12, 24, 36, 48, and 60 months post-operatively. Body mass index (BMI) and weight were measured at each follow-up visit. Also, the presence or absence of obstructive sleep apnea (OSA), type 2 diabetes mellitus (T2DM), gastroesophageal reflux disease (GERD), hypertension (HTN), and hyperlipidemia were recorded.

All statistics were run through SigmaPlot statistical software. Descriptive statistics were used to analyze preoperative characteristics such as age, weight, height, and body mass index (BMI). Calculations were made to determine their %EWL and percentage excess BMI loss (%EBMIL). Categorical variables were analyzed using the chi-squared test, Fisher’s exact test, or Student’s *t* test for quantitative and qualitative variables. We used Student’s *t* test for mean comparisons between the two groups (NLSG vs BSLG). The comparison was also made between banded and non-banded superobese patient population by using Student’s *t* test. Data were collected in the form of mean ± standard deviation. For all analyses that involved inferential statistics, a *P* value < 0.5 was considered statistically significant.

Additionally, complications from each patient were also recorded. For analysis, they were divided into those that occurred within the first 30 days and those that occurred subsequently. Additionally, they were divided into minor and major complications.

## Operative Technique

All operations were done laparoscopically. We begin the dissection on the greater curvature 3–4 cm from the pylorus. Dissection is continued until the left crus of the diaphragm is well visualized. Resection is started about 3–4 cm from the pylorus over a 40-F gastric calibration tube up to the angle of Hiss. A gastric sleeve of less than 100 ml in volume remains. No staple line reinforcement was done.

**Fig. 1:**
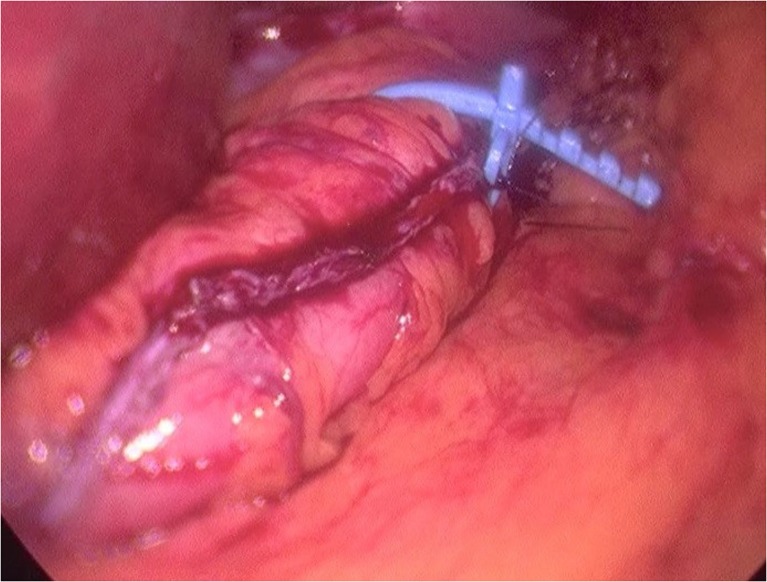
Intraoperative image of banded sleeve gastrectomy

A silastic ring (MiniMizer Ring®) is placed 4–5 cm from the gastroesophageal junction. The atraumatic needle of the Minimizer ring is introduced behind the sleeve through the lesser omentum in between the vessels of the lesser curvature. It is closed according to the manufacturer’s instruction and fixed with two non-resorbable sutures. Ring circumference of 6.5 or 7 cm are used for females, and 7 or 7.5 cm are used for males. The placement of the ring added less than 5 min to the operation. To avoid the damage to the posterior wall of the stomach, it is essential that the gastric calibration tube is inside at the moment of the ring closure and that there is 5 mm space between the ring and the pouch upon closure. No bleeding or damage to the gastric wall was noted (Fig. 1).

## Results

Of 147 patients, 51 underwent NLSG and 96 underwent BLSG. Patients in the NLSG group were a little older than in the BLSG group (*P* = 0.002). Similarly, male to female ratio was also significantly different between the two groups (*P* = 0.04), with BLSG group having more males than females. Demographic characteristics and rates of comorbidities are seen in Table 1. Of 147 patients, 112 patients had completed 1 year, 79 patients had completed 2 years, 56 patients had completed 3 years, 36 patients had completed 4 years, and 30 patients had completed 5 years of follow-up. Follow-up was possible for 99 patients after 1 year (88.3%), 75 patients after 2 years (94.9%), 46 patients after 3 years (82.1%), 30 patients after 4 years (83.3%), and 25 patients after 5 years (83.3%).

**Table 1:** Demographics

	NLSG	BLSG	*P* value
N	51	96	
Age	54.8 ± 14.1	47.9 ± 12.2	*0.002*
M/F	22/29	60/36	*0.04*
Weight	125.7 ± 25	129.7 ± 27.4	0.38
BMI	44.9 ± 7	43.7 ± 7.3	0.33
EBW	64.8 ± 19.8	62.8 ± 21.3	0.6
Band length	–	6.8 ± 0.3	
Comorbidities			
Diabetes mellitus	12	12	0.13
Hypertension	17	22	0.24
Sleep apnea	13	32	0.42
Hypercholesterolemia	11	19	0.96
Depression	1	1	0.77

(*P* < 0.001 or 0.05 is considered statistically significant). Statistically significant values are presented in Italic form

Abbreviations: *n*, number of patients; IBW, ideal body weight; EBW, excess body weight, NLSG, non-banded laparoscopic sleeve gastrectomy; BLSG, banded laparoscopic sleeve gastrectomy

## Weight Loss Analysis

The weight loss data were categorized into two sections as demonstrated in Table 2. Percentage excess BMI loss (%EBMIL) and %EWL in the BLSG group was higher than in the NLSG group and had a statistically significant difference at each given time point. At 1 year, the patients had an average %EBMIL of 72.3 ± 29.7 and 91.4 ± 25.2 in NLSG and BLSG, respectively (*P* = 0.001). Similarly, %EWL at 1 year was 60.6 ± 21.8 and 77.4 ± 20.5 in NLSG and BLSG, respectively (*P* < 0.001).

**Table 2:** Excess BMI loss (EBMIL) and excess weight loss (EWL) between the entire subset

	NLSG (*N* = 51)			BLSG (*N* = 96)				
	*N* (%)	EBMIL (%)	EWL (%)	*N* (%)	EBMIL (%)	EWL (%)	*P* value between EBMIL	*P* value between EWL
3 m	46/50 (92%)	38 ± 14.6	32.4 ± 10.8	89/96 (92.7%)	47.2 ± 20.7	40.3 ± 15.6	*0.008*	*0.003*
6 m	43/47 (91.4%)	56.5 ± 20.6	47.2 ± 15	83/88 (94.3%)	70.1 ± 22.7	59.2 ± 17.8	*0.001*	*< 0.001*
9 m	38/41 (92.6%)	67.8 ± 28.5	56.6 ± 20.3	73/81 (90.1%)	85.6 ± 24.4	71.8 ± 18.6	*< 0.001*	*< 0.001*
12 m	35/41 (85.3%)	72.3 ± 29.7	60.6 ± 21.8	64/71 (90.1%)	91.4 ± 25.2	77.4 ± 20.5	*0.001*	*< 0.001*
24 m	34/37 (91.8%)	74.9 ± 31.5	61.8 ± 23.2	41/42 (97.6%)	91.1 ± 21.5	77.4 ± 16.3	*0.01*	*0.001*
36 m	25/31 (80.6%)	70.5 ± 32	59 ± 23.6	21/25 (84%)	96.7 ± 18.1	83.3 ± 12.7	*0.002*	*< 0.001*
48 m	17/20 (85%)	67.8 ± 32	58.3 ± 23.6	13/16 (81.2%)	100.2 ± 19	86.2 ± 11.7	*0.003*	*< 0.001*
60 m	15/17 (88.2%)	66 ± 32.8	57.8 ± 25	10/13 (76.9%)	102.4 ± 19.3	86.7 ± 11.9	*0.004*	*0.003*

Abbreviations: n, number of patients; NLSG, non-banded laparoscopic sleeve gastrectomy; BLSG, banded laparoscopic sleeve gastrectomy. Statistically significant values are presented in Italic form

**Fig. 2:**
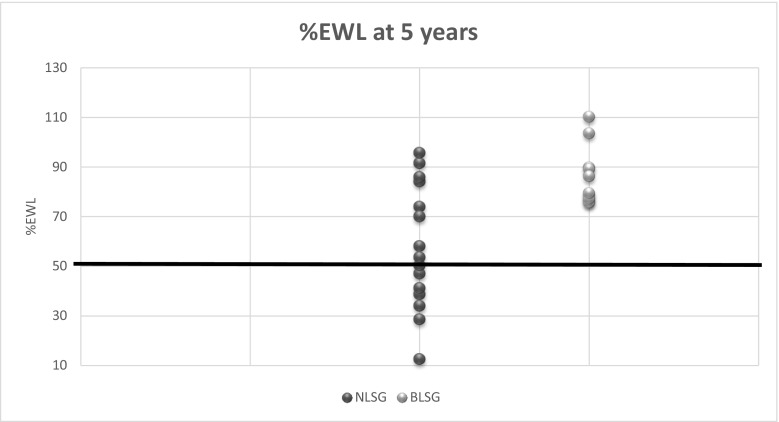
%EWL at 5 years follow-up. Data are displayed as the %EWL achieved by each patient at the 5-year follow-up visit in the NLSG and BLSG group. A bold line indicates the %EWL of 50%: 35.2% of NLSG patients had %EWL < 50% and 0 of BLSG patients had %EWL < 50%. Abbreviations: NLSG = non-banded laparoscopic sleeve gastrectomy, BLSG = banded laparoscopic sleeve gastrectomy

At 5 years, the patients had an average %EBMIL of 66 ± 32.8 and 102.4 ± 19.3 in NLSG and BLSG, respectively (*P* = 0.004). Similarly, %EWL at 5 years was 57.8 ± 25 and 86.7 ± 11.9 in NLSG and BLSG, respectively (*P* = 0.003). These results clearly show that the %EBMIL and %EWL decrease over time in the NLSG group, while both parameters continue to increase over time in the BLSG group with a statistical significant difference between two groups. Figure 2 shows the distribution of %EWL for both groups at the 5 years follow-up visit. These results show that in the NLSG group, 35.2% of the patients have < 50%EWL at the 5 years follow-up, whereas none of the BLSG-treated patients had < 50%EWL.

**Fig. 3:**
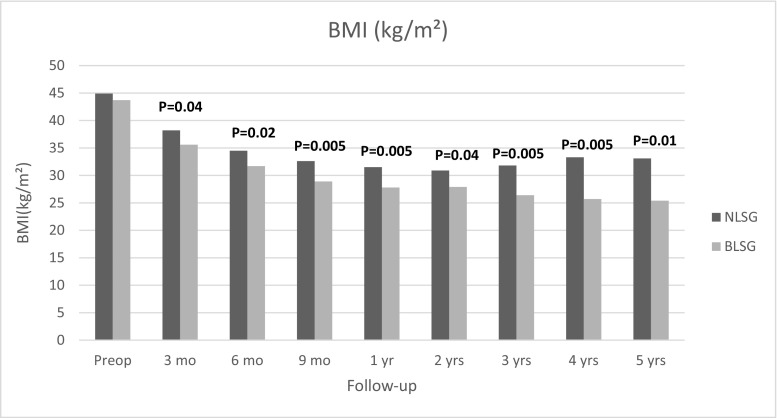
Evolution of BMI data (kg/m^2^). Data are displayed as a mean BMI in kg/m^2^ during the study period. BMI decreased more in the BLSG group with statistical significant difference at every given time point. Abbreviations: NLSG = non-banded laparoscopic sleeve gastrectomy, BLSG = banded laparoscopic sleeve gastrectomy

The mean BMI at 12 months was decreased from pre-op 44.9 ± 7 kg/m^2^ to 31.5 ± 7.2 kg/m^2^ in NLSG group, whereas in BLSG group BMI decreased from 43.7 ± 7.9 kg/m^2^ to 27.8 ± 5.5 kg/m^2^ at the 12 months follow-up visit. Additionally, over the next 4 years, BMI decreased more in BLSG group than in NLSG group with a statistical significant difference at each follow-up visit (Fig. 3).

## Weight Regain

The BLSG group had less weight regain (2%) at the 5-year follow-up visit compared to NLSG group (19.6%) (*P* < 0.001). After 5 years in the NLSG group, 80.3% had no increase in BMI points compared to 97.9% in the BLSG group. In the NSLG group, 12% of the patients had an increase of less than 5 BMI points compared to 2% in the BLSG group, while 8% of the NLSG group had an increase of more than 5 BMI points compared to 0 patients in BSLG group (Table 3).

**Table 3:** Weight regain

	NLSG (*N* = 51)	BLSG (*N* = 96)	*P* value
Weight regain*	1.8 ± 2.8	0.34 ± 0.4	
< 5 pts. BMI	6 (12%)	2 (2%)	
> 5pts BMI	4 (8%)	0	
Total	*10* (*19.6*%)	*2* (*2*%)	*P* < *0.001*

*Weight regain was measured in the number of BMI points (lowest BMI achieved-BMI at 5 years). Values are expressed mean ± standard deviation

Abbreviations: n, number of patients; NLSG, non-banded laparoscopic sleeve gastrectomy; BLSG, banded laparoscopic sleeve gastrectomy. Statistically significant values are presented in Italic form

## Weight Loss Analysis in Superobese NLSG and BLSG Patients

Table 4 shows the %EBMIL and %EWL between the superobese NLSG and BLSG group, respectively. Although the difference was not statistically significant, the BLSG group consistently outperformed the NLSG group in terms of both these parameters: at 5 years, superobese BLSG patients had 81.9 ± 1.6% EBMIL and 78.3 ± 1.6% EWL compared to 67.1 ± 29.4% EBMIL and 61.6 ± 24.6% EWL in the NLSG group.

**Table 4:** Excess BMI loss (EBMIL) and excess weight loss (EWL) between superobese non-banded and banded population

	Superobese non-banded (*N* = 11)			Superobese banded (*N* = 16)				
	*N* (%)	EBMIL (%)	EWL (%)	*N* (%)	EBMIL (%)	EWL (%)	*P* value between EBMIL	*P* value between EWL
3 m	11/11 (100%)	24.3 ± 10.5	23.9 ± 4.8	15/16 (93.7%)	29.4 ± 8.4	28.5 ± 9.2	0.1	0.1
6 m	10/11 (90.9%)	42.9 ± 17.6	40.2 ± 14.3	15/16 (93.7%)	47.3 ± 12	44 ± 12.3	0.4	0.4
9 m	9/10 (90%)	50.6 ± 21.1	48 ± 19.2	13/15 (86.6%)	59 ± 14.1	55.2 ± 14.7	0.2	0.3
12 m	8/10 (80%)	61.6 ± 24.6	58.5 ± 21.5	13/14 (92.8%)	68.4 ± 15.8	63.8 ± 16	0.4	0.5
24 m	8/9 (88.8%)	69 ± 26.7	63.2 ± 23	11/11 (100%)	74.8 ± 17.2	70.6 ± 17.4	0.5	0.4
36 m	8/9 (88.8%)	68.3 ± 28	62.3 ± 24.2	6/8 (75%)	84.9 ± 14.2	80.4 ± 13.3	0.2	0.1
48 m	7/7 (100%)	63.3 ± 31.3	58.2 ± 26.8	3/4 (75%)	84.5 ± 4.7	78.4 ± 1.2	0.2	0.2
60 m	6/6 (100%)	67.1 ± 29.4	61.6 ± 24.6	2/3 (66.6%)	81.9 ± 1.6	78.3 ± 1.6	0.5	0.3

Abbreviations: n, number of patients; NLSG, non-banded laparoscopic sleeve gastrectomy; BLSG, banded laparoscopic sleeve gastrectomy

## Complications

The complication rate was higher for the BLSG group (14.5%) compared to the NLSG group (9.8%) (Table 5). However, most of the complications seen within the BLSG group were late and minor. A total number of early complications seen with BLSG group were 3: Two patients with post-operative bleeding and 1 patient with an abscess. All the 3 complications appeared in the perioperative period. There was no early complication in the NLSG group. A total number of late complications seen with BLSG group were 11 (11.4%): seven patients with vomiting (7.2%), and 4 patients with ring-related problems (4.1%). All the late complications seen within the BLSG group were minor. Late complications seen within the NLSG group were 5 (9.8%): four patients with vomiting (7.8%) and 1 patient needed revision to bypass for insufficient weight loss and diabetes (1.9%). Ring-related problems seen within the BLSG group were the following: four patients with a functional stenosis at the level of the ring; 3 needing ring enlargement to 7.5 cm and 1 patient needing ring removal. There were no difference in the episodes of dysphagia between the BLSG group and the NLSG group in the first post-operative year. However, there was more difference in the dysphagia in the following years between the 2 groups, with the BLSG group having more episodes. The exact level of dysphagia is hard to quantify since these patients adapt their eating pattern to their specific level of gastric restriction. Most of the patients do not complain about this because of the fear of weight regain in case of loss of restriction.

**Table 5:** Complications

	NLSG (*N* = 51)	BLSG (*N* = 96)
Early minor	–	–
Early major	–	Post-op bleeding-2
		Abscess-1
Total early complications	0	3 (3.1%)
Late minor	Vomiting-4 (7.8%)	Vomiting-7 (7.2%)
		Ring-related problems-4 (4.1%)
Late major	Omega bypass-1 (1.9%)	–
Total late complications	5 (9.8%)	11 (11.4%)
Total overall complications	5 (9.8%)	14 (14.5%)

Abbreviations: n, number of patients; NLSG, non-banded laparoscopic sleeve gastrectomy; BLSG, banded laparoscopic sleeve gastrectomy

## Discussion

Despite the fact that the LSG has gained tremendous popularity worldwide, the durability remains a major concern. Insufficient weight loss and weight regain in the mid-term follow-up as well as in some long-term follow-up has been described [5]. One of the major reasons for this failure is pouch dilation. There are many reasons for the gastric pouch dilatation, including technical error during the operation. The superior pouch dilation may occur because of an incomplete release of the posterior gastric fundus or preservation of a part of the fundus to avoid injury of the esophagogastric junction or when the last stapler is fired > 1 cm away from the gastroesophageal (GE) junction. On the other hand, an inferior pouch dilatation may rise due to antral preservation, which may occur due to the misplacement of the bougie or misidentification of the pylorus [16]. Another possibility for antrum dilation is when the stomach is resected > 4 cm distance from pylorus [17].

Literature has shown that there has been an improved weight loss in vertical gastroplasty and RYGB with an additional circular reinforcement of a gastric pouch [13, 18]. So, why would an additional circular reinforcement improve weight loss in LSG? To answer this question, one has to observe the mechanism of laparoscopic adjustable gastric banding (LAGB) and LSG separately and with its combined effect. It has been found that weight loss after LAGB is mainly due to satiety and not restriction [19]. While LSG does not have much effect on satiety compared to other bariatric procedures, thus implanting an additional circular reinforcement in LSG would improve this effect. Additionally, satiety is also increased due to a slow food transportation in the longitudinal part of the sleeve due to continued restriction [20]. At the same time, the ileal break mechanism will be triggered due to the fast transit of food bolus into the small intestine. All these effects combined improve the weight loss in BLSG.

Several prosthetic devices and materials have been used for weight loss surgery, including linea alba, fascia lata, meshes, porcine, and bovine grafts; however, the most commonly used ring is a silastic ring (e.g., Minimizer® or GaBP ring™). The minimizer ring has an advantage over other rings because of the easy placement and closure and the intraoperative flexibility allowing adjustment to the desired diameter. Ease of placing the ring is assisted by a blunt, silicone covered introduction needle that simplifies retro gastric placement (11). Additionally, it forms a pseudo-capsule which does not easily incorporate in scar tissue and is easily removable [13].

In this current study, overall weight loss after NLSG is within the range mentioned in the literature. The position statement issued by the ASMBS on LSG has shown an EWL after LSG ranges between 53 and 69% with a tendency for weight regain [21]. Bohdjalian et al. reported 55% EWL with 19.2% weight regain at 5 years [7]. In this currents study, NLSG patients quickly lost 60.6% EWL at year 1; however, further weight loss after year 1 was not significant and had some weight regain in the following years (%EWL dropped from 61% at 2 years to 57% at 5 years). However, the BLSG group had increased %EWL at each follow-up visit. %EWL at 1 year after BLSG was 77.4% which was again increased to 86.7% at 5 years. These results indicate that additional banding does not only increase %EWL in early post-operative years but continues to do so in late post-operative years as well. Similar to %EWL, %EBMIL showed a significant difference in the BLSG group at each follow-up visit (Table 2).

Apart from %EWL and %EBMIL, the interesting thing to note is the difference between BMI and weight regain during the 5-year period. Although the starting BMI for each group was similar (43.7 vs 44.9), the evolution of BMI data shows a divergence between both groups with BLSG patients showing more decrease in BMI post-operatively (25.4 vs 33.1) with a statistically significant difference (Fig. 3). Weight regain in BMI points at 5 years shows a statistical difference with NLSG showing considerably greater weight regain compared to BLSG (Table 3). At 5-year follow-up, only 2% of the BLSG patients had weight regain when compared to 19.6% of NLSG patients (19.6%) (*P* < 0.001). Some may argue that the impressive difference in the weight regain might be a result of frequent dysphagia in this group. However, as we discussed earlier, the exact level of dysphagia is hard to objectify, and thus it is difficult to conclude if this is one of the reasons for higher weight loss or less weight regain with the BLSG group. Our results also showed that there are almost 35 times as many patients (35.7%) in the NLSG group that end up below 50% EWL bracket after 5 years than in the BLSG group (Fig. 2). These results show that additional banding also helps in maintaining weight loss and reducing weight regain at long-term follow-up. This is also supported by the fact that removal of the ring causes increases in weight [22]. Since the weight loss in the BLSG group is more sustainable, we would expect this group to show improved metabolic results, but we have not measured this. Literature shows very divergent weight loss results with BLSG. Fink, et al. also found that weight loss after BLSG was greater than NLSG at 3 years (66.74% vs 55.95% EWL) [23]. While Tognoni et al. [12] did not find any statistically significant difference in weight loss between two groups at 1 year. Karcz et al. published matched cohort analysis of 25 BLSG (using Minimizer ring®) patients and 25 patients with LSG. He found no difference in %EWL at 12 months. [11].

In spite of promising outcomes, LSG has poor results in patients with BMI over 50 and has a large standard deviation [5]. Criticism could be made that a ring might work well for the obese LSG population but might not work for the superobese LSG patient population (BMI > 50). Agarwal et al. [24] reported the first case report of BLSG as a primary procedure on the super obese patient. This study did not evaluate the weight loss data, but the surgery was performed successfully with no intraoperative or early post-operative complications. In the current study, we compared the weight loss outcomes between superobese BLSG and NLSG patients. Though there was no statistically significant difference in weight loss between the superobese BLSG and NLSG patients, the weight loss was certainly higher in the BLSG group as compared to the NLSG group (Table 4). At 5 years, %EWL in the superobese NSLG group was only 61.6% compared to 78.3% in the superobese BLSG group. That means BLSG has better weight loss in patients with BMI < 50 as wells as BMI > 50 when compared to NLSG patients.

Beside insufficient weight loss, band-related complications are the major throwbacks for the fading popularity of LAGB. On the other hand, we cannot compare the ring with the adjustable band. The adjustable band causes restriction by compressing the stomach wall, while the ring for the sleeve only prevents dilatation. In this study, 4 patients in the BLSG group had ring-related complications. Three patients had functional stenosis at the level of the ring, which was corrected by ring enlargement to 7.5 cm. These 3 patients then had full resolution of their symptoms. One patient also complained about too much restriction. The ring was enlarged with 0.5 cm; however, he continued to have complaints and finally had the ring removed. Amazingly, this patient was still complaining of too much restriction while X-ray studies and gastroscopy showed a normal sleeve without signs of stenosis or torsion. An advantage of the MiniMizer Ring® over other rings is that one can enlarge it, or make it smaller. Ring enlargement is done on an out-patient basis with 3 or 4 trocars of 5 mm. In this study, we did not see any ring erosions, slippage or migrations. This is explained by the fact that the ring does not compress the stomach wall. Only when the food bolus is passing there is a temporary compression. This inhibits the patients of eating too fast. The same results were shown by Alexander et al., who did not note a high migration incidence either [25]. Alexander et al. used AlloDerm® rings, which have the tendency to stretch over time and allow a relatively quick passage of higher volumes of food. Karcz et al. [15] observed two Minimizer® ring-related vomiting and needed ring removal (8%). Symptoms resolved immediately after ring removal. Mason et al. [26] have performed many vertical banded gastroplasties (VBG) using a Marlex ring. Although Marlex rings have a higher incidence of strictures, he did experience few ring-erosions requiring ring removal. Fink et al. [23] experienced 3 ring removals (7.1%) due to severe regurgitation. The most important thing to note in his study is that he did not see an increased ring removal rate in the longer follow-up period. Stubbs et al. found that most of the ring removal after banded LRYGB (BLRYGB) is associated with the ring size and he recommended increasing the ring size from 5.5 to 6.5 cm, to avoid these complications [27]. In our study, we have used 6.5 to 7 cm for all the females and 7 to 7.5 cm for all the males. With larger ring sizes, we expect lower incidence rate of ring-related problems. All the studies suggest that, although the ring-related problems can be challenging to patients as well as to surgeons, the reported incidence after BLSG is 4% in our study and can be resolved without sequels. And though there is a potential risk of band migration and band slippage after BLSG, we have not encountered this complication in the literature as well as in the present study.

For every advantage, there is a cost. In this study, the BLSG group had higher complication rates compared to NLSG group. Since the complications were different in the two groups, it was difficult to compare them except the frequency of vomiting which was higher in BLSG group but not statistically significant (*P* = 0.83). However, this trend is also seen in other published series on BLSG. Karcz et al. observed 40% frequency of vomiting in BLSG at 1 year [15] and some of these symptoms initiated by pouch enlargement. We had 7 patients (7.2%) with late frequency of vomiting that required only conservative management. In patients complaining of too much restriction, we proposed an enlargement of the ring, but most of these patients refused this because of the fear of weight regain. Reflux remains a major concern before and after LSG. The mechanism of LSG on reflux is still debatable. Some propose that LSG can cause or worsen the GERD [28, 29], while others believe that obesity is a risk factor for GERD and when one loses weight with LSG, one improves the symptoms of GERD [30]. One of the limitations of this study is that we did not routinely scope our patients for GERD or evaluated the patients with GERD questionnaire, and this is the main reason we do not have exact numbers describing this complication in this study. Many patients do not complain about GERD unless we asked them about taking proton pump inhibitors. However, we did have some patients in both groups that complained of GERD, but there was no significant difference between two groups. But without the GERD symptom score, it is difficult to comprehend this difference between two groups. The studies in the past have shown the ring implantation do not have any relevant impact on new-onset reflux. On the contrary, it tends to improve the reflux in patients with pre-existed reflux [23].

We have performed banded LRYGB (BLRYGB) since 2004 and recently published a prospective cohort study comparing BLRYGB with LRYGB [13] at 5 years. The results between two groups showed a similar trend to the results of this current study, i.e., the banded group had more weight loss, and less weight regains at 5 years compared to the non-banded group. At 1 year, BLRYGB had EWL of 75.2% while our results with BLSG showed EWL of 77.4%. However, at 5 years, BLRYGB had EWL of 74%, while BLSG had EWL of 86.7%. Even the weight regain at 5 years with BLRYGB was 1.2 ± 1.5 BMI points while with BLSG was 0.34 ± 0.4 BMI points. The comparison of BLRYGB with BLSG suggest that both groups lose similar excess weight at 1 year; BLSG has better weight loss than BLRYGB at long term with minimum weight regain and band-related complications.

The main limitation of this study is the sample size. Although the sample size for BLSG is 96 and for NLSG is 51, the number of patients at each year is relatively low, with a decreasing number of patients each year, the difference in weight loss of the BLSG compared to the NLSG at 5 years has been impressive and statistically significant. The other limitation of the study was the lack of quality of life (QOL), satiety questionnaire, and GERD questionnaire data. These questionnaires would have helped to compare the complications more accurately and further the understanding on the mechanism of the banding the proximal stomach. That being said, it is important to note that this is the first paper with long-term outcomes for BLSG patients with a silicone ring. This is also the first study that shows the long-term outcomes of BLSG in severely obese patients. Most importantly, no severe ring-related complications were shown in the study.

## Conclusion

In summary, the results from this study show that BLSG was more effective in reducing and maintaining weight compared to the NLSG group at 5 years. More than 97% of the patients in the BLSG group had no weight regain at all, compared to the NLSG group, where only 80% of the patients had no weight regain, but 8% had an increase in BMI of more than five points 5 years following LSG surgery. Furthermore, the NLSG group had 35.2% of patients who had less than 50% EWL at 5 years follow-up compared to 0 patients in the BLSG group. This advantage of BLSG comes with a cost of higher late complication rates compared to the NLSG group; however most of these complications were minor and acceptable. There was no ring erosion, slippage, or migration seen with BLSG patients. BLSG surgery was found to be safe and effective in maintaining weight loss on the long term compared to the NLSG group with low incidence of band-related problems. Prospective comparative studies with large sample size are needed to further validate our results.
